# Osteosarcoma: a comprehensive review

**DOI:** 10.1051/sicotj/2017028

**Published:** 2018-04-09

**Authors:** Amirhossein Misaghi, Amanda Goldin, Moayd Awad, Anna A Kulidjian

**Affiliations:** 1 Department of Orthopaedic Surgery, University of California San Diego San Diego CA USA; 2 School of Medicine, University of Jeddah Jeddah Saudi Arabia

**Keywords:** Osteosarcoma, Review, Oncology, Sarcoma, Tumor

## Abstract

Osteosarcoma (OS) is a relatively rare tumor of bone with a worldwide incidence of 3.4 cases per million people per year. For most of the twentieth century, five-year survival rates for classic OS were very low. In the 1970s, the introduction of adjuvant chemotherapy in the treatment of OS increased survival rates dramatically. The current article reviews the various types of OS and analyzes the clinical and histological features. We also examine historical and current literature to present a succinct review of methods for diagnosis and staging, as well as treatment, and we also discuss some of the future directions of treatment.

## Introduction

Osteosarcoma (OS) is a primary malignant bone tumor with a worldwide incidence of 3.4 per million people per year [1]. For most of the twentieth century, five-year survival rates for classic OS were 20%. In the 1970s, the introduction of adjuvant chemotherapy in the treatment of OS increased survival rates to 50% [2–4]. Before the mid-1970s, amputation was the routine treatment for high-grade OS. By 1990, the management of high-grade OS shifted to include more emphasis on chemotherapy and limb salvage. The current survival rate has increased to >65% [5].

## Epidemiology

OS is a rare sarcoma that has the histological findings of osteoid production in association with malignant mesenchymal cells [6]. OS is the third most common cancer in adolescence, with only lymphomas and brain tumors being more prevalent, and with an annual incidence of 5.6 cases per million children under the age of 15 [7–9]. Peak incidence is in the second decade of life [10, 11]. Before the age of five, OS is rare [12]. OS arises sporadically, with few cases associated with known inherited defects in cell cycle regulation, but about 70% of tumor specimens demonstrating a chromosomal abnormality. These commonly involve mutations in tumor-suppressor genes or in DNA helicases [13].

## Types

The World Health Organization’s histologic classification of bone tumors divides OS into central, intramedullary, and surface tumors, with a number of subtypes under each group [14].

### Central

#### Conventional osteosarcoma

Conventional OS is the most common type of OS and represents 80% of all osteosarcoma cases primarily affecting individuals in the first and second decades of life. It can be subdivided into osteoblastic, chondroblastic, and fibroblastic groups depending on the predominant features of the cells; there are no significant differences in clinical outcomes among these categories [14]. OS is typically high grade and originates in the intramedullary cavity. On radiographs OS can be osteolytic or osteoblastic, or both. Eighty percent of cases are located in the metaphysis of long bones, but OS can also arise in the diaphysis of long bones as well as the axial skeleton [15]. On histology, evidence of bone or osteoid production by the tumor cells is a requirement for diagnosis [16].

#### Telangiectatic osteosarcoma

Telangiectatic osteosarcoma (TOS) accounts for 4% of OS [17]. Histologically, dilated blood-filled cavities and high-grade sarcomatous cells on the septae and peripheral rim characterize TOS. Radiographically, TOS is metaphyseal, with geographic patterns of bone destruction and a wide zone of transition. Moth-eaten or permeative destruction can be seen [18]. It is important to differentiate TOS from aneurysmal bone cysts (ABC) on imaging. ABC is described as an eccentric lytic lesion with a “blown-out” space in the bone. The two lesions are known to appear radiographically similar and cases of mistaken TOS for ABC have been reported [19–21]. Although it was believed that the prognosis of TOS is worse than the Conventional Type, recent studies suggest that there is no difference between the two types [22, 23].

#### Small-cell osteosarcoma

Small-cell osteosarcoma (SOS) constitutes 1–2% of all OS. The histological features of SOS show cells that are small, and have round hypochromatic nuclei with little nuclear polymorphism, similar to Ewing’s sarcoma [24, 25]. However, while the production of osteoid by tumor cells confirms the diagnosis for OS, it is not a characteristic feature of Ewing’s sarcoma [26]. A destructive process with lytic areas and sclerosis is found on radiographs [25].

#### Low-grade osteosarcoma

Low-grade osteosarcoma (LOS) accounts for 1–2% of all OS. However, LOS generally affects persons in the third or fourth decade of life [17, 27]. LOS can be difficult to recognize, as it is low grade and may resemble parosteal osteosarcoma, fibrous dysplasia, or desmoplastic fibroma [28]. While there is a risk of transformation to conventional OS if treated with curettage alone, the prognosis is significantly better in LOS [29].

### Surface

#### Parosteal osteosarcoma

Parosteal osteosarcoma (PAOS) is a low-grade osteosarcoma that originates from the periosteum. PAOS represents 4–6% of OS and commonly affects the posterior aspect of the distal femur. It may also occur in other sites including the proximal humerus and proximal tibia [30, 31]. Radiographs demonstrate a densely ossified and lobulated mass, while the medullary cavities are spared [32]. Histologically, PAOS exhibits streams of bone trabeculae that show a high degree of parallel orientation, similar to what may be seen in a periosteal new bone reaction [26].

#### Periosteal osteosarcoma

Periosteal osteosarcoma (PIOS) has a matrix component that is mainly cartilaginous and less common than parosteal. PIOS tends to arise between the cortex and the cambium layer of the periosteum, and therefore a periosteal reaction is usually visible on radiographs [33]. On histopathologic examination, an intermediate-grade tumor is seen, containing a cartilaginous matrix with areas of calcification [26].

#### High-grade surface osteosarcoma

High-grade surface osteosarcoma (HGSOS) constitutes less than 1% of all OS [17] and manifests as a surface lesion with a high-grade appearance histologically [34]. Local growth is accelerated in HGSOS more than in parosteal osteosarcoma. HGSOS has the same malignant potential as the conventional type, therefore some degree of localized invasion of the cortex and endosteum may be seen [26]. Radiographically, HGSOS demonstrates a surface lesion with partial mineralization, and the tumor may extend to surrounding soft tissues [35].

## Diagnosis and staging

The technology and techniques used to diagnose osteosarcoma have improved over the past several decades [36]. For any suspected bone lesion, a preoperative imaging protocol should be followed, which includes taking at least two X-ray views of the whole bone and the adjacent joint. Radiographs will show an ill-defined lesion arising at the metaphysis of bone, with osteoblastic and/or osteolytic areas, periosteal reaction, and a soft tissue mass [37–40].

Magnetic resonance imaging (MRI) is warranted to evaluate the lesion’s invasion into the soft tissue and neurovascular structures, level of bone marrow replacement, skip lesions, and extension into the bordering joint [37–41]. In 1994, Schima et al. investigated the usefulness of contrast-enhanced MRI in determining intra-articular tumor extension in patients with OS. Ten of 46 patients had extension of tumor into the joint on pathology, of which all were preoperatively identified on MRI. Eleven other tumors showed potential intra-articular extension on MRI, providing a sensitivity of 100% and a specificity of 69%. Pathologically, 12 patients were found to have transphyseal involvement, of whom all had preoperative evidence of transphyseal involvement on MRI, providing a sensitivity and specificity of 100%. The authors concluded that T1-weighted contrast-enhanced imaging is useful for detecting intra-articular OS involvement [39]. In 1991, Shuman et al. showed that fat-suppression MRI allowed exposure of a greater quantity of abnormal tissue than proton density and T2-weighted imaging; yet, they suggested that fat suppression may cause fat planes to appear ambiguous, which can cause difficulty in precise staging of disease. They did not use contrast-enhanced T1-weighted images in this study [42].

Computed tomography (CT) scans are useful in defining cortical irregularities, fracture sites, mineralization, and neurovascular involvement [37–40]. Bone scintigraphy can help show polyostotic involvement, metastases, and intraosseous tumor extension. Angiography may aid in showing vascular anatomy. This is helpful for preoperative planning in patients with tumors at the proximal tibia or shoulder girdle, as these are areas with common vascular anatomic anomalies [39, 40]. Positron emission tomography (PET) scans can be used to assess the primary lesion(s) and to detect metastatic lesions in other bones and the lungs [37]. Some suggest using PET scans to assess the histologic response of the disease to chemotherapy as well as to predict progression-free survival [40]. CT and/or X-ray of the chest should be completed to assess pulmonary metastases, as this is the most common location of metastatic disease in osteosarcoma [36, 37, 39, 40, 43]. It is reasonable to obtain a repeat CT scan 6–12 weeks following the first, as it is difficult to detect metastatic lesions that are less than 5 mm in size [40].

A biopsy is essential in the diagnosis of OS. Eventual tumor resection must include the biopsy tract, as this tract could get contaminated with tumor cells. The surgeon should choose a biopsy tract that will be included in this future surgery [38]. Preferably, the surgeon performing the biopsy would be the same surgeon to ultimately perform the resection; if this is not possible, an experienced radiologist may perform the biopsy with imaging assistance, or an experienced surgeon may perform a percutaneous biopsy without radiologic guidance [40, 44]. Open biopsy was once considered the gold standard due to its accuracy rate of 98%; however, there are associated complications with open biopsy, especially if the biopsy is conducted outside of the ultimate treatment facility [45]. Core biopsy is preferred because there is less risk of local contamination. This is important in patients who may have limb-sparing surgery [37, 39]. Hau et al. set to determine whether CT-guided biopsy is as accurate as open biopsy in diagnosing musculoskeletal tumors. They retrospectively reviewed 359 patients who had undergone CT-guided biopsy and found an overall accuracy of 71%; of these, 258 were CT-guided core biopsies and had an accuracy of 74%, while 101 were fine needle aspirations and had an accuracy of 63% [45]. They concluded that while both core needle and open biopsy provide adequate tissue for accurate analysis, fine needle aspiration should not be used, as it does not provide a large enough sample for a precise diagnosis [40, 45]. Welker et al. conducted a retrospective review of 173 core needle biopsy procedures, of which 90 were performed without radiologic guidance, and found that 88.2% of these biopsies were sufficient for diagnosis. In this study, percutaneous needle biopsy provided a positive predictive value of 100%, a negative predictive value of 82%, a sensitivity of 81.8%, and a specificity of 100%. They deemed percutaneous needle biopsy a safe and effective method for diagnosing musculoskeletal masses [44]. Similarly, the prospective study by Skrzynski et al. found an accuracy rate of 84% for patients undergoing a closed needle biopsy and found this to be an accurate and less expensive procedure [46].

After the biopsy is performed, a frozen section will sometimes be completed. Histologically, OS will appear as osteoblastic, chondroblastic, or fibroblastic [16, 47]. Many tumors will display aspects of all three cell types and matrix [16], and findings should be reviewed by a pathologist with experience in musculoskeletal pathology [39]. Mitsuyoshi et al. reviewed the biopsies of 157 patients and found that an experienced musculoskeletal pathologist was able to distinguish malignant from benign lesions with 100% accuracy in bone tumors, and obtain a specific diagnosis in 96% of the cases of bone tumors [48].

There is no laboratory test that is diagnostic for OS; however, complete blood count, basic metabolic panel, renal and liver function tests, and urinalysis are all useful to assess the patient’s baseline organ function prior to the start of chemotherapy. Osteoblastic activity can be assessed with alkaline phosphatase levels, and lactate dehydrogenase levels can be used to assess osteoclastic activity [38]. Recent studies have demonstrated that C-reactive protein (CRP) has prognostic value in OS, as patients with higher levels of CRP have a statistically higher probability of death due to disease. Funovics et al. demonstrated that patients whose preoperative CRP was greater than 1.0 mg/dL had a significantly worse prognosis than those with CRP levels below 0.02 mg/dL [49, 50].

The staging classification used in osteosarcoma is the Musculoskeletal Tumor Society staging scheme, also known as the Enneking system. This system establishes whether a tumor is low or high grade (I or II), whether the tumor is intra- or extra-compartmental (A or B), and whether any metastases are present (III). Stage IA represents a low-grade tumor that is intra-cortical, IB represents a low-grade tumor that is extra-cortical, IIA represents a high-grade tumor that is intra-cortical, and IIB represents a high-grade tumor that is extra-cortical. Metastatic disease automatically places the patient in the stage III category [38, 40, 41]. Most commonly, OS patients are diagnosed at stage IIB [38].

## Treatment

Conventional treatment for OS consists of a combination of neoadjuvant and adjuvant chemotherapy, and surgery [4, 51]. Prior to the use of chemotherapy, there was less than a 20% survival rate in high-grade conventional osteosarcoma even with surgical amputation, indicating the presence of micrometastases (typically pulmonary) prior to surgery [4, 52]. The low grade can typically be treated with excision alone and chemotherapy is avoided if final pathology confirms low grade.

### Surgical treatment

The goal of tumor surgery is complete resection of disease via wide excision of the tumor [53]. Surgical options can be divided into limb salvage versus amputation.

#### Limb salvage

Limb salvage surgical techniques provide a safe methodology of treatment for 85–90% of patients with OS [54–56]. There are two essential steps of limb salvage, including resection and reconstruction. Resection is crucial to the elimination of disease. It should include excision of previous biopsy sites and tracts with at least a 2 cm margin. All major vessels should be identified prior to ligation. Preoperative imaging, such as bone and CT scan, should be utilized to determine the necessary quantity of bone to be osteotomized. This should be around 6–7 cm distal to the lesion to ensure clear margins [57, 58]. Custom jigs generated by computers have also gained favor as a tool to improve accuracy in wide resection of OS [59–61]. Khan et al. conducted a study on six pairs of matched cadaver femurs utilizing computer-aided design software for one of the femurs and manual resection for the other. They found a significantly higher deviation from the preoperative plan in their manual femurs compared to the ones with custom jigs [61]. Computer-aided navigation can be especially useful in pelvic and sacral tumors, as it allows safe margins to be obtained as planned preoperatively, without over-resection of weight-stabilizing bone [59, 60]. Tumor resection surgery in patients who are skeletally immature brings up the issue of physeal destruction and the possibility of growth disturbances. Traditionally, location of the tumor through the growth plate was a contraindication for limb salvage and an indication to amputate. Now, current treatments include resection with expandable growth endoprosthesis, allograft endoprosthetic composites, or rotationplasty [62]. Resections around the joints are challenging, and joint contamination precludes limb preservation surgery, necessitating an amputation. Some centers advocate preservation of the joint through resections through the growth plate [53, 63]. In 1994, Canadell described a method of combining distraction osteogenesis with an external fixator with tumor resection in order to try and decrease growth discrepancies. They operated on 20 patients, of whom none had local recurrences and three had pulmonary metastases. They ultimately determined this method a safe and effective way to resect tumor but maintaining the epiphysis of long bones [63]. Other centers have simulated these results [53], however experience is limited.

Reconstruction is the next step in limb salvage. It should be noted that non-weight-bearing bones, such as the clavicle or proximal fibula, do not require reconstruction, as excision alone does not cause functional deficits [58, 64]. When reconstruction is utilized in weight-bearing bones, it can be divided into endoprosthetic replacement and biological reconstruction. Endoprosthetic replacement is a form of limb salvage reconstruction, and has been reported to have good functional outcomes and better cosmetic and psychological benefits in comparison to other forms of treatment, including amputation and rotationplasty [65]. The design of these implants includes modular, custom-made, and growing implants for the skeletally immature. Since the 1990s implant design has been modular with titanium segments and cobalt-chrome alloy tapers in order to prevent cold welding [66]. Titanium alloys are associated with a lower rate of late infections than cobalt-chrome alloys. Silver-coated titanium megaprostheses are thought to reduce infection rate further [67]. Iodine coating of titanium implants has also been shown to decrease infection risk [68]. The first endoprostheses were custom designed. Modular prostheses allow for use of off-the-shelf components that are less expensive and timely to make than custom-made, and have proven to have good survivorship [66, 69]. Ahlmann et al. retrospectively reviewed 211 patients who had undergone limb salvage with modular endoprosthesis, and found a survivorship rate of 78% at five years post op and 60% at 15 years post op, which generally outlasted the survival rates of the patients [69]. Schwartz et al. compared the survivorship of 85 patients with modular implants and 101 patients with custom-designed implants and found that there was 15-year survivorship of 93.7% and 51.7%, respectively [66]. Finally, in the case of children, expandable prosthesis can be utilized, which involves a prosthesis that allows for interval lengthenings via a series of minor surgical procedures. The growth plates of the affected bone are removed, and the prosthesis is lengthened by 1–2 cm per surgery, in order to correlate with the contralateral, healthy extremity [62].

Biologic replacement is the second form of limb reconstruction, which includes allograft, autograft, recycled autografts, and allograft prosthetic composite reconstructions. Massive bone allografts have been in use since as early as 1908, however debate regarding their effectiveness and durability continues. Donati et al. performed a retrospective review evaluating 92 patients who had undergone massive allograft reconstruction. Forty-five percent and 29% had an “excellent” and “good” outcome, respectively, while 15% of the allografts failed [70]. Similarly, Gebhardt et al. examined a cohort of 53 patients with allograft reconstruction, and of the 38 who did not have a recurrence of the disease there was a 70% satisfactory rate [71]. These authors concluded that overall bone allografts can be an effective method of tumor reconstruction after resection [70, 71]. Finally, allograft prosthetic composites (APC) combine implants with allograft for reconstruction. APC arthroplasty is utilized for weight-bearing joints, including the hip and knee. It combines the benefits of a biologic graft, including better reinsertion of soft tissues and preservation of anatomy, with the stability and ability to immediately weight bear of a prosthesis. It is at risk for nonunion and infection [70, 72].

Autografts can be used in a number of ways. The fibula is an ideal bone for autograft harvest, as it is long, tubular, relatively superficial, and minimally load sharing. It can be vascularized or not, however non-vascularized graft is dependent on the blood supply and bone quality it is placed into. Vascularized fibula tends to have a reduced time to union and faster hypertrophy than non-vascularized [73]. The autogenous recycling method involves tumor-bearing autografts being heated, frozen, or irradiated. Benefits of pasteurized autografts include preserving anatomy and bone-inductive activity, but complications include bone absorption, fracture, pseudoarthrosis, and infection [68, 74]. Tsuchiya et al. treated 33 malignant bone tumors with pedicle frozen autograft (by liquid nitrogen), and obtained excellent results in 75.7% of patients, but also had complications in 48%, including infection, fracture, and recurrence [68].

Some studies have reported slightly higher recurrence rates with limb salvaging compared to amputation, however the overall survival rate of patients who recur is comparable [75]. In fact, some studies show that survival rates are higher with limb salvage than amputation. In 2001, Ferrari et al. demonstrated an eight-year-survival rate of 62 in patients undergoing limb salvage, compared to 43% for those undergoing amputation [55]. Endoprosthetic replacement in tumor surgery has been shown to lead to improved quality of life. Lang et al. did a retrospective review to analyze the sporting abilities in 27 patients with OS who received limb-salvaging modular endoprostheses. They found that by five years post-operation, the same percentage of patients who played sports previously played sports post-operatively. They concluded that patients can reach high levels of sports after a modular endoprosthesis, and the potential to do so depends more on preoperative activity rather than the procedure and implant itself [76]. Many now consider limb salvage to be the preferred treatment for malignant sarcomas [77].

#### Amputation

Amputation, once the standard surgical treatment of OS, is now typically reserved to the non-resectable tumor with soft tissue and neuromuscular contamination not amenable to repair. Many studies argue that limb salvaging surgery provides better daily function than amputation and is equal, if not better, in terms of survival [62, 77–79]. A novel surgical treatment includes osteointegration implants, which are used as an adjunct to treatment in amputees to increase function. Branemark et al. conducted a prospective study of 51 patients who had undergone transfemoral amputations, either due to tumor or trauma. These patients had a survival rate of 92% at two years, and overall reported increased use of prosthetic and quality of life [80].

Rotationplasty involves resection of the distal femur, followed by rotation of the lower leg 180° thus turning the ankle joint into a “knee” joint [78, 81, 82], with the gastrocnemius and soleus plantar flexors becoming “knee” extensors [81]. It was first described in 1930 by Borggreve for a shortened leg after a patient had tuberculous ankylosis of the knee joint; however, it was not until 1974 that the procedure was described as a treatment for OS [78, 81]. Rotationplasty has shown good functional and rehabilitative results, especially in children and active adults [78, 81, 82]. However, its odd appearance causes psychological problems in some patients [65, 83].

Salzer et al. [78] conducted a study in 1981 with 15 patients who underwent rotationplasty for OS. Patients ranged from six to 32 years old and were followed from six to 63 months post-operatively. Three patients died of metastases at 24 and 25 months post-operatively, while 12 patients had no evidence of disease at the completion of the study. Functionally, all patients could fully extend their “knees” and achieved flexion between 70° and 90°. In 2015, Gradl et al. aimed to describe the long-term quality of life results of rotationplasty and in this study the patients overall had positive experiences after their rotationplasties [83].

### Chemotherapy treatment

Prior to the 1970s, chemotherapy was not used for osteosarcoma and survival rates were dismal. In 1972, MD Anderson released a study treating their osteosarcoma patients with chemotherapy and presenting a two-year survival rate of 50%. In 1981, a prospective trial began that compared the outcomes of 27 patients treated without any adjuvant chemotherapy to 32 patients receiving either Adriamycin, high-dose methotrexate, or a combination of bleomycin, Cytoxan, and actinomycin-D. In 1984, the trial was discontinued when it became clear that the patients receiving chemotherapy had a statistically significant advantage, as 55% remained disease-free at two years, in contrast to only 20% of the non-chemotherapy group disease-free. Survival at two years was also significant, with 80% versus 48% of the patients remaining alive in the treatment and control groups, respectively [4].

The standard of care for osteosarcoma is currently neoadjuvant chemotherapy, surgery, and adjuvant chemotherapy. The four chemotherapy agents that are in nearly all treatment regimens include methotrexate with leucovorin rescue, doxorubicin, cisplatin, and ifosfamide. Patients who have metastatic disease may also be treated with etoposide. Tumor necrosis response to neoadjuvant chemotherapy dictates the overall response to treatment. O’Kane et al. retrospectively reviewed 97 patients with osteosarcoma treated with chemotherapy and surgery, and found that those who had greater than 90% tumor necrosis had an 82% five-year survival rate, while those who had less than 90% tumor necrosis had a 68% five-year survival rate [52]. The idea of “dose intensity” is important as well. Imran et al. retrospectively reviewed 703 charts of patients with localized OS, and found that those who waited over 21 days after surgery to resume chemotherapy had a significantly higher mortality rate. The authors suggest restarting chemotherapy within the first 21 days post-operatively in order to maintain dose intensity [84].

### Radiation treatment

Radiation treatment has a controversial role in the treatment of OS due to its questionable effectiveness and associated risk of infection. An interesting application as an adjunct to low-cost reconstructive modality has been popularized in Japan. A 2013 study retroactively reviewed 101 patients with sarcoma (37 of whom had OS) after receiving extracorporeal irradiation (ECI) and yielded some promising results. The ECI consists of en bloc resection of the involved bone, treatment of each bone segment with 50 Gy radiation, and ultimate replantation of the bone. None of the 37 patients with OS had disease recurrence. The authors promote ECI as a low-cost treatment that is effective at preventing disease recurrence and carries a low risk of infection [51].

## Future directions

There have been no significant advances in the treatment of the disease over the last 10 plus years, however advancements are slowly being made in the treatment of OS as more is being understood about the pathophysiology of the disease. Novel drug delivery systems and immunotherapies are being developed, and old dogmas of neoadjuvant chemotherapy and surgical resection are being challenged. Jones et al. evaluated 24 patients with distal femoral OS who underwent MRI pre- and post-neoadjuvant chemotherapy, and determined that although neoadjuvant chemotherapy did affect surgeon planning of resection level, it did not do so in a reliable direction. With MRI’s obtained post-neoadjuvant chemotherapy, more surgeons elected to proceed with amputation, implying that neoadjuvant chemotherapy does not offer significant clinical benefit in the anatomical planning of resection level. A proposed theory as to the dogma of chemotherapy was noting that edema improves post-treatment, leading to the belief that chemotherapy improves resectability [85].

New reconstructive modalities are also being proposed by Tsuchiya et al. They have studied the long-term outcomes of utilizing liquid nitrogen (LN2) to kill tumor cells and replanting patient’s own tissue after LN2 treatment. They report on 17 osteosarcomas treated with a pedicle frozen autograft, with 14 cured of the disease, two local recurrences, and one leading to distal metastasis [68]. This treatment may offer a cost-effective perfectly matched autograft as an alternative to allografts and endoprosthesis, though long-term effectiveness needs to be further studied.

Advances in treatment are also being made on the molecular level. Mason et al. made promising strides in a canine model of OS, using an attenuated listeria vaccine to deliver and induce innate HER2/neu immunity, leading to a significant reduction in metastatic disease and increases in survival [86].

Tyrosine kinase inhibitors have been investigated, and *in vitro* studies have shown that they reduce motility, colony formation, and invasiveness of disease, and may be beneficial in managing cases of OS metastases [87].

## Summary

Osteosarcoma is a rare bone tumor found in areas of rapid bone turnover, most commonly the distal femur and proximal tibia of adolescent patients. Early on, treatment of osteosarcoma typically involved surgical resection in the form of amputation or reconstruction with auto- or allograft. With the addition of neoadjuvant chemotherapy to treatment protocols, five-year survival rates have dramatically increased. The current treatment of osteosarcoma involves neoadjuvant chemotherapy, wide resection, followed by adjuvant chemotherapy, with strict emphasis on the overall intensity of treatment and resumption of the post-resection chemotherapy as soon as possible. Progress is being made in the areas of immune therapy and targeted chemotherapy, and the investigation of newer treatment strategies has yielded promising early results.
